# “Delirium and COVID-19”: From Symptomatology to Laboratorial and Neuroimaging Findings”

**DOI:** 10.1192/j.eurpsy.2022.102

**Published:** 2022-09-01

**Authors:** D. Telles Correia, F. Novais

**Affiliations:** 1 FACULDADE DE MEDICINA UNIVERSIDADE DE LISBOA, Psychiatry, Lisboa, Portugal; 2 Lisbon University, Faculty Of Medicine, Lisbon, Portugal

**Keywords:** COVID-19, Delirium, Laboratory findings, neuroimaging findings

## Abstract

**Introduction:**

The infection caused by the SARS-CoV-2 virus called COVID-19 may affect not only the respiratory system but also the central nervous system (CNS). Delirium is a frequent and serious condition in COVID-19 patients and may be caused by the direct invasion of the CNS or the induction of CNS inflammatory mediators or by indirect effects due to the systemic inflammatory status, other organ failure, prolonged mechanical ventilation time, immobilization but also social isolation. We aim to critically review literature reporting this syndrome in patients infected by the SARS-CoV-2 virus with a particular emphasis on reported clinical, laboratorial and neuroimaging findings. Methods: A state-of-the-art literature review was performed using PubMed, Embase and Web of Knowledge using the following keywords: delirium, COVID-19, SARS-Cov-2, neuroimaging, laboratorial findings. Results: More than 50% of patients with COVID-19 may present with delirium and in about 20% of the cases this is the primary presentation of the disorder. Previous data suggests that these patients may show a higher frequency of certain symptoms such as agitation, myoclonus, abulia, and alogia. Some distinct neuroinflammatory syndromes have been identified in patients presenting with delirium associated with the virus, namely, autoimmune encephalitis, Acute Disseminated Encephalomyelitis (ADEM) and stroke showing its potential for CNS involvement. Many of these patients present normal brain imaging, EEG and CSF findings but others have more specific laboratorial changes such as elevated creatinine kinase, elevated D-dimer levels, abnormal coagulation parameters and positive SARS-Cov-2 PCR in CSF or meningeal enhancement, ischemic stroke and perfusion changes in MRI imaging.

**Disclosure:**

No significant relationships.

